# Protective Effects of Proline–Rich Peptide in a Rat Model of Alzheimer Disease: An Electrophysiological Study

**DOI:** 10.15412/J.BCN.03080101

**Published:** 2017-01

**Authors:** Naser Khalaji, John Sarkissian, Vergine Chavushyan, Vaghinak Sarkisian

**Affiliations:** 1. Department of Physiology, School of Medicine, Uremia University of Medical Sciences, Uremia, Iran.; 2. Orbeli Institute of Physiology, National Academy of Sciences of the Republic of Armenia, Yerevan, Republic of Armenia.

**Keywords:** Hypothalamic Proline–Rich Peptide (PRP-1), Alzheimer disease, Amyloid-β, Hippocampus

## Abstract

**Introduction:**

Alzheimer disease (AD) is the most common form of dementia in the elderly that slowly destroys memory and cognitive functions. The disease has no cure and leads to significant structural and functional brain abnormalities. To facilitate the treatment of this disease, we aimed to investigate proline-rich peptide (PRP-1) action of hypothalamus on hippocampal (HP) neurons and dynamics of their recovery, after intracerebroventricular (ICV) injection of amyloid-β (Aβ).

**Methods:**

Experiments were carried out on 24 adult, male Albino rats (average weight: 230±30 g). The animals were randomly divided into 3 groups (control, Aβ, and Aβ plus PRP-1). Electrophysiological patterns of hippocampal neurons in response to stimulation of entorhinal cortex (EC) with high frequency stimulation (50 Hz) were studied.

**Results:**

It was found that Aβ (25–35) suppresses the electrical activity of hippocampal neurons. The PRP-1 would return this activity to normal levels.

**Conclusion:**

In general, PRP-1 has protective effect against AD-related alterations induced by amyloid peptides. This protective effect is probably due to stimulation of the immune and glia system.

## 1. Introduction

Alzheimer disease (AD) is a progressive neurodegenerative disease that causes dysfunction of mental activities such as learning and memory in the elderly (Meamar, 2013; Rissman, De Blas, & Armstrong, 2007). Neurodegenerative diseases are characterized by progressive dysfunction and death of cells in selected areas of the nervous system, associated with protein apoptosis. Intermediate compounds such as oligomers and protofibrils have cytotoxic effects on neurons. The main basic processes were caused by genetic, environmental, endogenous factors, and abnormal protein dynamics (Jellinger, 2009).

The main cellular pathogenesis of AD is extracellular sedimentation of amyloid-β (Aβ) plaque and intracellular collection of hyperphosphorylated microtubule-associated protein that assembled in neurofibrillary plexus. This accumulation produced neurodegeneration and synaptic dysregulation (Hardy & Selkoe, 2002). According to the amyloid hypothesis, the progressive accumulation of soluble Aβ (40–42) oligomers will result in cholinergic dysfunction and mild cognitive impairment in the first stage of the disease (Walsh & Selkoe, 2004), in later stages, it advances to Aβ plaque deposition, neurofibrillary tangle accumulation, chronic inflammation, neurodegeneration, and consequent severe dementia (Blennow, de Leon, & Zetterberg, 2006).

Aβ is a 39 to 43 amino acid peptide and a major component in the neuritic plaques of AD (Bond et al., 2003). It is believed that full-length Aβ peptide is responsible for AD. The interesting property of Aβ peptide is its various segments (including 1–28, 25–35, and 34–42) which show similar biophysical and biochemical properties like full-length Aβ (1–42) peptide (Shanmugamo & Lavarapu, 2004).

Electrophysiological and molecular studies performed in animal models of AD have shown that cognitive impairment is related to considerable alterations in the synaptic plasticity (Balietti et al., 2012) and synaptic depression induced by accumulation of Aβ (Hsieh et al., 2006).

In the pathogenesis of AD, intracellular Ca^2+^ homeostasis disturbance plays a central role (Khachaturian, 1994) as its increase in intracellular concentration triggers mechanism for amyloid plaque formation and neurofibrillary deposition (Stutzmann, 2007). It was shown that Aβ can disrupt neuronal Ca^2+^ homeostasis and increase ion permeability, associated with the formation of artificial ion pores in lipid membranes or activation of endogenous ion channels on the cell surface (Small et al., 2009). In other words, Aβ can modulate ion channels (including the voltage-dependent Ca^2+^ and potassium channels), which reinforces the influx of the ion into the cell, forming specific Ca^2+^ conducting pores and modulating the N-methyl-D-aspartate (NMDA) and nicotinic receptors (Carafoli & Brini, 2000).

Electrophysiological studies have documented that neurodegenerative diseases alter long-term potentiation (LTP) and long-term depression (LTD) in the hippocampus and other regions of the temporal lobe (Kumar, 2011).

The deficits of cholinergic or noradrenergic systems characterizing AD have significant role in the glial activation and local inflammatory processes (Carnevale, De Simone & Minghetti, 2007). Recently, it was demonstrated that astrocytes provide processing and integration of the synaptic information as well as controlling synaptic transmission and plasticity (Perea, Navarrete, & Araque, 2009). Moreover, astrocytes have functional receptors for neurotransmitters and response to stimulation through excretion of gliotransmitters (Rossi & Volterra, 2009).

Despite important advances in medicine and large financial investments, the neurobiological bases of cognitive impairment in AD and its treatment are not fully understood. Therefore, we studied the effect of PRP-1 in a rat model of AD induced by Aβ (25–35). PRP-1 was first synthesized by Dr. A. A. Galoyan in the Institute of Biochemistry, NAS republic of Armenia and kindly provided to us for the study. This compound has several biological effects on immune and nervous system, including protective effects on neurodegenerative processes (Galoyan et al., 2010). In the present study, we used a new mathematical program with the possibility of averaging the entire mass of experimental data.

## 2. Methods

Experiments were carried out on 24 adult male Albino rats (230±30 g body weight). The animals were randomly divided into 3 groups; control (3 μL normal saline, ICV injection), Aβ (Aβ [25–35] 3 μL 10 M solution, ICV injection), and Aβ plus PRP-1 (0.1 mg/kg PRP-1, IP, every day since the next day after injection of Aβ, for 3 weeks).

All the processes were accomplished by the “Principles of Laboratory Animal Care” (NIH publication No. 85-23, revised 1985), as well as the specific rules provided by the Animal Care and Use Committee of National Medical and Health Service.

During acute phase of experiments, the animals were immobilized by 1% dithylinum (25 mg/kg, IP) and under artificial ventilation the spinal cord (SC) at T1–T3 level (with ultrasound scalpel) was dissected to achieve encephale isole. After removing skin of cranium, the trepanation of skull was performed from bregma, until lambda and dura mater was removed. This procedure was done on the stereotaxic apparatus. Stereotaxic (stereotaxic apparatus CЭЖ-1 made in the experimental workshop of the Institute of Physiology, Ukrainian Academy of Sciences) orientated glass electrodes of 1–2 μM tip diameter were filled with 2 M NaCl and inserted into hippocampal (HP) fields of CA1, CA3 for recording single-neuron spikes flow activity evoked by high frequency stimulation (HFS) of ipsi-lateral entorhinal cortex (EC) (rectangle current pulses of 0.05 ms, 0.08–0.16 mA, and frequency of 50 Hz, during 1 second). Later, recordings were performed 12 weeks after bilateral ICV injection of normal saline into control group, and 12 weeks after bilateral ICV injection of Aβ (25–35) (3 μL of 10 M solution) into Aβ group. Also, recordings were performed 12 weeks after bilateral ICV injection of Aβ with systemic use of PRP-1 (0.1 mg/kg IP, every day since next post operation day for 3 weeks).

Stimulating and recording electrodes were put according to the stereotaxic coordinates of the rat atlas (Paxinos & Watson, 2005), EC (AP −10.0, L ±3.5, DV +4.0 mm), HP (AP −3.5; L ±3.5; DV +2.8–4.0 mm). Post stimulus activity was revealed as tetanic potentiation (TP) and tetanic depression (TD) followed by post tetanic potentiation (PTP) and post tetanic depression (PTD). After recording, the pulse flows were analyzed by a special mathematical program before and after stimulation, for getting “raster” of single-neuron prestimulus and poststimulus spike flows in real time.

Then, basis on the number of prestimulus histograms of sum spikes, raster, and frequency histograms were arranged. By means of selected comparable groups of neuronal spiking, the similar complex averaged pre-stimulus histograms and frequency histograms were constructed. This computer program allows the separation of stimuli, superposed on action potentials (AP) during their close succession in the process of TP and TD and avoids traditional complex intra-cellular recording approach of long-term TP and TD.

To determine the statistical significance of differences during inter spike intervals as well as before and after the stimulus, we used nonparametric criterion, 2-sample Mann-Whitney U test for the testing of 2 independent samples. Since the number of recorded spikes was over than several hundred spikes within 10 seconds after stimulation, we used a variant of this test, taking into account its asymptotic normality, i.e. Z-test. Comparison of critical values with the tabulated values of the normal distribution at significant levels of 0.05, 0.01, and 0.001 (for different trials), showed that as a result of HFS for most samples of neuronal activity, spiking had a statistically significant change with a minimum significance of 0.05.

## 3. Results

The study on impulse activity flow of single hippocampal neurons evoked by HFS (50 Hz) of EC during 1 second was carried out in Wistar rats in the control, Aβ, and Aβ plus PRP-1 groups. In general, we recorded 94 neurons, which are shown in the following 3 Figures.

The results of following 3 Figures were demonstrated by pre-stimulus and frequency histograms of sum spikes, constructed raster of prestimulus and poststimulus excitatory TP and TD effects, and manifestation of spike activity of single-neuron of hippocampus to HFS of EC in the real time.

**Figure 1:**
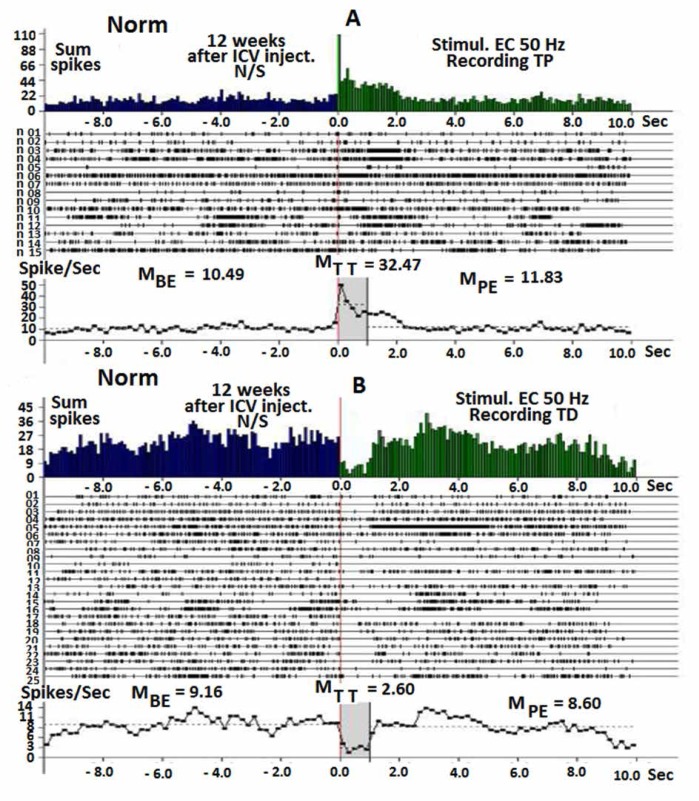
**A, B.** Prestimulus histograms of sum spikes (from above), constructed rasters of pre stimulus and poststimulus excitatory - TP (A) and depressor - TD (B) effects of single neurons of hippocampus to HFS (50 Hz) of EC during 1 second in the real time (10 seconds before and after) stimulation, 12 weeks after injection of normal saline in control group. Bottom diagram of neurons spikes summarized frequency, presented in the raster and in the real time, indicating average digital values of 10 seconds before excitation (M_BE_) and 10 seconds after excitation (M_PE_) and during 1 second of tetanic time (M_TT_).

The flow of single-neuron spikes activity was analyzed under TP and TD recording, with subsequent PTP and PTD, caused by HFS of EC. As shown in Figure 1, the control group produced TP and TD of hippocampal neurons 12 weeks after ICV injection of normal saline during stimulation of EC with HFS (50 Hz) (Figure 1. A, Figure 1. B). TP almost exceeded 3 times the level of background (Figure 1. A), and TD decreased 3.5 times lower than of background activity (Figure 1. B). There was a correlation between the activity of hippocampal neurons before and after excitation.

**Figure 2:**
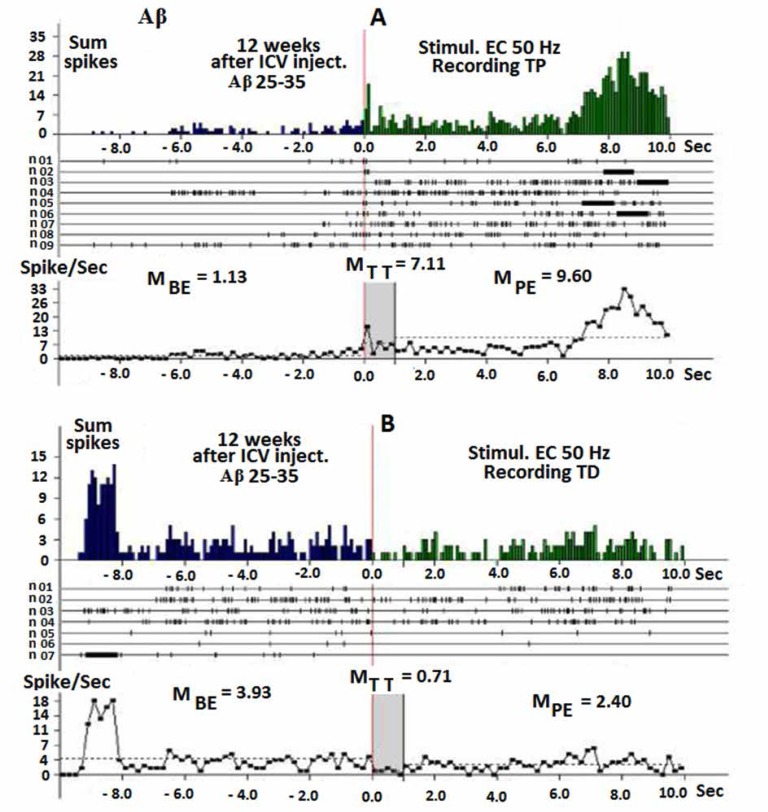
**A, B.** Prestimulus histograms of sum spikes (from above), constructed rasters of pre stimulus and poststimulus excitatory - TP (A) and depressor - TD (B) effects of single neurons of hippocampus to HFS (50 Hz) of EC during 1 second in the real time (10 seconds before and after) stimulation, 12 weeks after injection of Aβ (25–35). Bottom diagram of neurons spikes summarized frequency, presented in the raster and in the real time, indicating average digital values of 10 seconds before excitation (M_BE_) and 10 seconds after excitation (M_PE_) and during 1 second of tetanic time (M_TT_).

Aβ group produced TP and TD in hippocampal neurons like that of the control group 12 weeks after ICV injection of Aβ (25–35) during HFS (50 Hz) of EC (Figure 2. A, B), but the number of spikes was fewer than that of the control group. There were no difference between left and right sides (before and after excitation) and administration of PTP (Figure 2. A). TP increased 6.29 times before excitation (Figure 2. A), and TD decreased 5.53 times lower than that before excitation (Figure 2. B).

**Figure 3:**
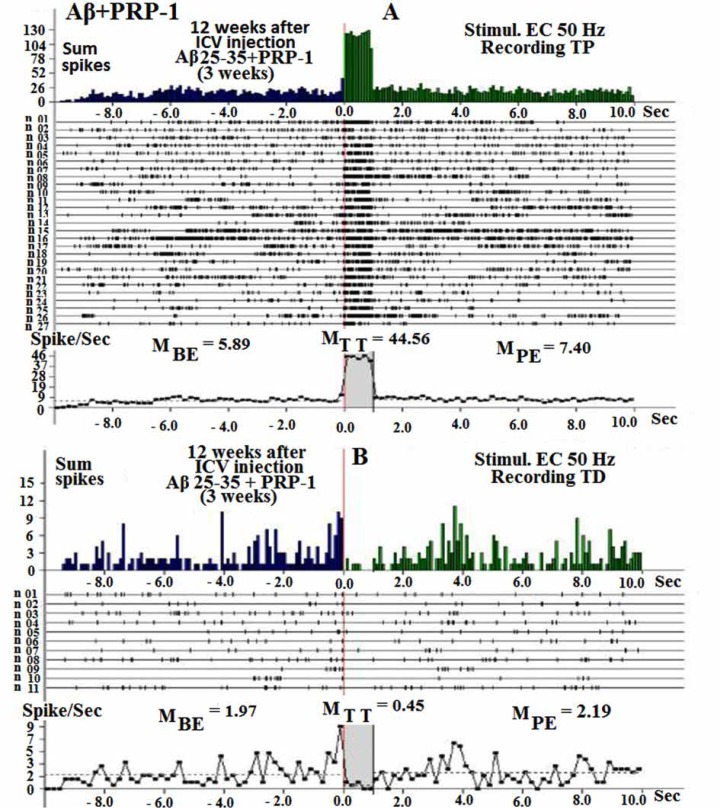
**A, B.** Prestimulus histograms of sum spikes (from above), constructed rasters of pre stimulus and poststimulus excitatory - TP (A) and depressor - TD (B) effects of single neurons of hippocampus to HFS (50 Hz) of EC during 1 second in the real time (10 seconds before and after) stimulation, 12 weeks after injection of Aβ (25–35) along with using PRP-1. Bottom diagram of neurons spikes summarized frequency, presented in the raster and in the real time, indicating average digital values of 10 seconds before excitation (M_BE_) and 10 seconds after excitation (M_PE_) and during 1 second of tetanic time (M_TT_).

As shown in Figure 3, in the group receiving amyloid with PRP-1 after 12 weeks, electrical activity of hippocampal neurons approached near to the activities of the control group. There was a correlation between the activities of hippocampal neurons before and after excitation.

Therefore, the results of this research showed that administration of PRP-1 leads to the return of inhibitory and excitatory poststimulus (early and late) activities of hippocampal neurons to normal levels in AD produced by Aβ (25–35).

## 4. Discussion

Study of reversible impairments of synaptic memory mechanisms in preclinical AD is deficient (Rowan et al., 2003). However, to identify AD mechanisms, it is necessary to study the synaptic plasticity or post tetanic manifestations of activity in the hippocampus and EC. These studies help identify the target of early therapeutic intervention (Mattson & Chan, 2003).

The results of this study in the control group demonstrated that, stimulus of EC with HFS produces TP and TD in the hippocampal neurons, and also there is electrical activity of these neurons before and after excitation. The EC is located in the medial temporal lobe or memory system of hippocampus and comprises the main portal between the hippocampal formation and the neocortex. It is in conjunction with the hippocampal formation (Witter, 2011). In AD, the earliest cell loss occurs in the entorhinal region of the medial temporal lobe (Rowland & Pedley, 2010). Regarding the dynamics of AD development 12 weeks after bilateral ICV injection of Aβ (25–35), it was found that the electrical activity of hippocampal neurons was suppressed; furthermore PTP and PTD appeared (Figure 2).

AD is a neurodegenerative disease that is characterized by abnormal mental functions such as learning and memory deficits (Rissman & Blas, 2007). One of the main causes of neurodegeneration is accumulation of β-amyloid plaques in the brains of patients with AD (Porayette et al., 2009). The Aβ peptide is derived from the amyloid precursor protein (Witter, 2011). Our study is supported by the fact that Aβ induces impairments in synaptic plasticity along with inhibition of hippocampal LTP and enhancement of long-term depression (Chen et al., 2013).

On our model of AD induced by 12 weeks bilateral ICV injection of Aβ (25–35) along with PRP-1 administration, TP and TD approached to normal level (Figure 3).

In this research, the excitatory effects due to Aβ (25–35) were successfully restored to normal level, which suggests protective action of PRP-1, irrespective of disease seriousness in AD model of rats.

The presented dominant depressor effect on hippo-campal neurons in sham-controlled and PRP-1 treated groups in terms of toxic effects of Aβ (25–35) suggest that generation of GABA by activation of the neurotransmitter system amino acids glutamine-glutamate-GABA is one of the methods of plastic reconstruction.

Based on the results of a recent study, regaining inhibitory poststimulus events, plays an important role in neurodegenerative disorders. It induces a protective effect, particularly on synaptic plasticity during preclinical stage (Shineman & Fillit, 2009; Hermes et al., 1996; Jensen, Jensen & Lambert, 1999; Tighilet & Lacour, 2001; Owens & Kriegstein, 2002).

Importantly, during development of nervous system in some systems, GABA acts as a trophic factor influencing various events, including proliferation, migration, differentiation, maturation of the synapse, cellular death, and expression of GABA_A_ receptors (Owens & Kriegstein, 2002). In AD, degeneration of hippocampal glutamatergic and intermediate brain acetyl cholinergic neurons may occur and preserve GABAergic neurons, expecting that inhibition is the protective mechanism for the neurons (Rissman, Blas & Armstrong, 2007; Palop et al., 2007).

Recently, a new regulatory mechanism was proposed through which GABAergic inhibitory post synaptic potentials occur in cultured hippocampal neurons (Jensen, Jensen & Lambert, 1999). However, the involvement of GABAergic inhibition during TD cannot be excluded in the present study.

Finally, it should be noted that oligomeric and protofibril forms of Aβ by attacking the neuronal and glial functions may be considered as strong blockers of LTP (LaFerla, Green, & Oddo, 2007; Walsch et al., 2002). In fact, AD pathogenesis could be linked to disturbances of synaptic mechanisms, particularly tetanic and posttetanic manifestations of activity in hippocampal region and EC (Mattson & Chan, 2003). Disorder of synaptic plasticity, especially LTP, is an early biochemical alteration in AD (Larson et al., 1999).

Thus, the present study showed that, besides controlling function, PRP-1 helps return the excitatory post-stimulus pattern of HP neurons activity to the norm, in an animal model of AD. However, the protective effect of PRP-1 in the early and late excitatory phases (on AD models induced by Aβ (25–35) showed minimal changes in inhibitory reactions. This may be due to treatment in the early preclinical stage of AD that does not require more active involvement of inhibitory assistance in the capacity of protector. Furthermore, at later periods of clinical behavior, receptors of the excitatory neuromediators seem to be more affected.

Recently a family of new PRP-1, produced by neurosecretory cells of hypothalamic paraventricular and supraoptic nuclei, was found. Many studies reported that PRP-1 has several biological actions on immune and nervous system, as well as common protective effect against neurodegenerative processes, astrocytes, and interleukin mechanisms (Galoyan et al., 2010; Galoyan, 2001). It is shown that PRP-1 normalizes the metabolism of phospholipids, increases the activity of creatine kinase, protein biosynthesis and activity of caspase 2 and 6, and leads to the activation of immune cells and production of cytokines (IL-1a, IL-1b, IL-6, TNF-a). PRP-1 has neuroprotective properties in the spinal cord hemi-section, exposed to snake venoms of Vipera raddei and Naja oxiana, after cutting and crushing of peripheral nerves (Galoyan et al., 2010; Galoyan, 2001). Recent studies demonstrated that the astrocytes can provide processing, transmission, and plasticity of synaptic neurons (Perea, Navarrete, & Araque, 2009).

In summary, during Aβ (25–35) administration, the possible GABAergic nature of the poststimulus depression (recorded in this study and supported by PRP-1) counteracts the neurodegeneration and provides a protective effect. This protective effect is likely to stimulate the immune plasticity of synaptic and glia systems.
